# Serum Copeptin Rises After Tolvaptan for Hyponatraemia, but Does Not Predict Risk of Rapid Sodium Rise: Pre‐Specified Secondary Analysis of the TVFR Trial

**DOI:** 10.1111/cen.70184

**Published:** 2026-08-03

**Authors:** Annabelle M. Warren, Kay Weng Choy, Rudolf Hoermann, Nicholas Russell, Mathis Grossmann

**Affiliations:** ^1^ Department of Endocrinology Austin Health Heidelberg Victoria Australia; ^2^ Department of Medicine, Austin Health University of Melbourne Heidelberg Victoria Australia; ^3^ Department of Endocrinology and Diabetes Alfred Health Melbourne Victoria Australia; ^4^ Northern Pathology Victoria, Northern Health Epping Victoria Australia

**Keywords:** arginine vasopressin (AVP), copeptin, hyponatremia (hyponatraemia), syndrome of inappropriate antidiuresis (SIAD, SIADH), tolvaptan

## Abstract

**Objective:**

Hyponatraemia is a common electrolyte disorder often driven by excess arginine vasopressin (AVP). Copeptin is a stable surrogate marker co‐secreted with AVP. It is unclear whether treatment of hyponatraemia with tolvaptan, an AVP‐V2 receptor antagonist, impacts copeptin. We aimed to assess the effects of tolvaptan on serum copeptin, compared to fluid restriction.

**Design:**

Pre‐specified secondary analysis of an open‐label randomised trial comparing tolvaptan or fluid restriction for 3 days.

**Patients:**

Hospitalised patients with plasma sodium (pNa) 115–130 mmol/L at a single‐centre tertiary hospital in Melbourne, Australia.

**Measurements:**

Copeptin measured at baseline and completion (Day 4, or discharge if sooner).

**Results:**

Copeptin results were available in 45/54 participants, randomised to tolvaptan (*n* = 25) or FR (*n* = 20). Mean baseline copeptin was 10.4 pmol/L. pNa increased in both groups, significantly more with tolvaptan as previously reported. Copeptin remained stable after FR, but significantly increased after tolvaptan (mean adjusted difference between groups over 3 days 8.4 pmol/L, 95% CI 2.1–14.6, *p* = 0.01). Baseline copeptin did not predict rapid sodium rise.

The rise in copeptin after tolvaptan may represent an exaggerated response to osmolality rise in these patients (‘reset osmostat’), or feedback mechanisms from AVP blockade.

**Conclusion:**

Tolvaptan increased serum copeptin compared to fluid restriction. Further research is required to determine if there is clinical utility for measuring copeptin in hyponatraemia before it is adopted into practice.

**Trial Registration:** ACTRN12619001683123.

## 1 Introduction

Hyponatraemia is the most frequent electrolyte disorder, affecting around 15% of hospital inpatients. It can be associated with significant morbidity and mortality [1]. Many causes of hyponatraemia, including the syndrome of inappropriate antidiuresis (SIAD), are driven by arginine vasopressin (AVP) which promotes water retention, resulting in hypotonic hyponatraemia [1].

Copeptin is a 39‐amino acid glycosylated peptide that is cleaved from the precursor protein of AVP. It is therefore a byproduct of AVP production co‐secreted in equimolar amounts, but more stable in serum. Hence, copeptin serves as a surrogate marker for AVP [2].

Currently, copeptin is not routinely assessed in hyponatraemia as it is not useful in differentiating between the most common causes or in guiding management [3]. Copeptin concentrations are typically elevated in SIAD but are also raised due to ‘appropriate’ AVP release with circulatory volume depletion (e.g., hypovolaemia, heart failure, liver failure), despite decreasing osmolality [4, 5]. The degree of elevation of copeptin does not reliably differentiate between these aetiologies [6].

First line therapy for SIAD is fluid restriction (FR) [7]. Second line therapies include tolvaptan (an AVP‐V2 receptor antagonist), urea and sodium‐glucose cotransporter 2 inhibitors (SGLT2i) [1, 7, 8]. The impact of SIAD therapies on copeptin concentrations is not well understood. Preliminary data have observed a rise in copeptin following treatment with SGLT2i in SIAD [9], and a rise in copeptin following ingestion of urea in healthy controls [10].

There are no available data about the impact of treatment of hyponatraemia with tolvaptan on copeptin. Given that approximately 20% of patients with moderate‐profound hyponatraemia treated with tolvaptan have overly rapid increase in plasma sodium (> 10 mmol/L) [11, 12, 13], it would be useful to identify these patients in advance, to ensure adequate monitoring and timely intervention if required.

We conducted a randomised clinical trial comparing two therapies for hyponatraemia: tolvaptan and FR. In addition to plasma sodium, symptom and safety outcomes, we aimed to compare serum copeptin response as a pre‐specified secondary outcome. We hypothesised that copeptin would rise more markedly with tolvaptan compared to FR. We also speculated that higher baseline copeptin might predict a higher risk of sodium overcorrection after tolvaptan.

## 2 Materials and Methods

This investigator‐initiated, 3‐day, randomised, open‐label trial occurred at a single tertiary hospital, Austin Health, in Melbourne, Australia. The trial received ethics approval from the Austin Health Human Research Ethics Committee (HREC/48055/Austin‐2019) and was prospectively registered (ACTRN12619001683123). Results of the primary outcome have been published [11].

We included hospitalised adults with pNa 115–130 mmol/L. Exclusion criteria included hypovolaemia, severe symptoms of hyponatraemia (e.g., seizure, coma), recent thiazide diuretic and others as detailed in the protocol [14]. Enroled participants were randomly assigned 1:1 to tolvaptan or FR.

Tolvaptan‐assigned patients were prescribed 7.5 mg oral tolvaptan on Day 1 and counselled to drink freely to thirst. Participants allocated to FR restricted their fluid intake below 1000 mL/day. Titration of each intervention occurred daily based on pNa change, up to maximum tolvaptan dose 30 mg/day; or FR limit below 500 mL/24 h [14]. Repeat direct pNa was checked 6 and 12 h post‐intervention to monitor rate of change. There was a pre‐specified protocol for IV 5% dextrose if pNa targets were exceeded, to prevent pNa rise > 10 mmol/L/24 h [11, 14].

Copeptin was measured at baseline (Day 1) and study completion (Day 4, or on discharge if this occurred earlier than Day 4). Serum from these timepoints was stored at −60°C by Austin Health Pathology Clinical Trials service. Copeptin measurement was performed using the BRAHMS Copeptin proAVP Kryptor assay from Thermo Fisher Scientific at Northern Pathology Victoria.

Baseline data are reported as mean (SD), and between‐group differences were tested with the Welch *t*‐test. Main analysis was by intention to treat, estimating in a repeated‐measures mixed‐effects model the mean adjusted difference between the tolvaptan and FR group over time, as previously reported in detail [11]. Models included strata from randomisation, baseline variable, time point, treatment group, interaction of time by group (treatment effect) and a random effect by subject. A two‐sided *p* value of < 0.05 was considered indicative of statistical significance. All statistical analyses were performed in the R statistical environment (version 4.4.0 for Mac) [15] with the additional packages lme4 1.1‐35.3 and effects 4.2‐2 [16, 17].

## 3 Results

A total of 54 participants were enroled and randomised. Copeptin results were available for 45 participants (20 randomised to FR, 25 to tolvaptan). Baseline characteristics of this group are shown in Table 1. The mean age was 79.6 years (SD 11.8) and 26 (57.7%) were female. The cause of hyponatraemia was deemed SIAD in 39 (87%), unknown in 8.8% and heart failure in 4.4%. Mean baseline pNa was 123.6 mmol/L (SD 3.47).

**Table 1:** Baseline characteristics of trial participants with copeptin levels measured.

	Fluid restriction (*N* = 20)	Tolvaptan (*N* = 25)	All (*N* = 45)
Age (years)	83.3 (11.2)	76.68 (11.6)	79.6 (11.8)
Sex, female	14 (70%)	12 (48%)	26 (57.8%)
Ethnic origin			
European/White	17 (85%)	22 (88%)	39 (86.7%)
Asian	2 (10%)	3 (12%)	5 (11.1%)
Multiracial	1 (5%)	0	1 (2.2%)
Cause of hyponatraemia			
SIAD – idiopathic	4 (20%)	11 (44%)	15 (33%)
SIAD – CNS pathology	3 (15%)	6 (24%)	9 (20%)
SIAD – malignancy	4 (20%)	1 (4%)	5 (11%)
SIAD – lung pathology	5 (25%)	5 (20%)	10 (22%)
Unknown	3 (15%)	1 (4%)	4 (9%)
Heart failure	1 (5%)	1 (4%)	2 (4%)
Volume status			
Euvolemic	19 (95%)	22 (88%)	41 (91%)
Hypervolemic	1 (5%)	3 (12%)	4 (9%)
Weight (kg)	61.3 (12.3)	68.3 (15.2)	65.3 (14.3)
Baseline biochemistry			
Plasma sodium (pNa)	123.8 (3.5)	123.5 (3.5)	123.6 (3.5)
Serum osmolality (mOsm/kg)	264 (11.1)	264 (7.7)	264 (9.3)
Urine sodium (mmol/L)	61.7 (35.8)	65.7 (32.2)	64.0 (33.5)
Urine osmolality (mOsm/kg)	444 (174)	429 (147)	436 (158)

*Note:* Values are *N* (%), mean (standard deviation).

The mean baseline copeptin was 10.4 pmol/L (SD 11.7) across the cohort. This was significantly higher in participants randomised to tolvaptan than FR (13.1 vs. 7.0 pmol/L, *p* < 0.001) by random effect (Table 2).

**Table 2:** Serum copeptin at baseline and Day 4 (or discharge if sooner).

	Fluid restriction (*n* = 20)	Tolvaptan (*N* = 25)	Mean adjusted difference (95% CI)	*p*
Copeptin (pmol/L)				0.01*
Day 1 (baseline)	7.0 (3.8)	13.1 (15.0)	—	
Day 4 (or discharge)	7.0 (4.1)	21.4 (19.3)	8.4 (2.1‐14.6)	

*Note:* Values are mean (standard deviation).

* *p* < 0.05.

**Figure 1:**
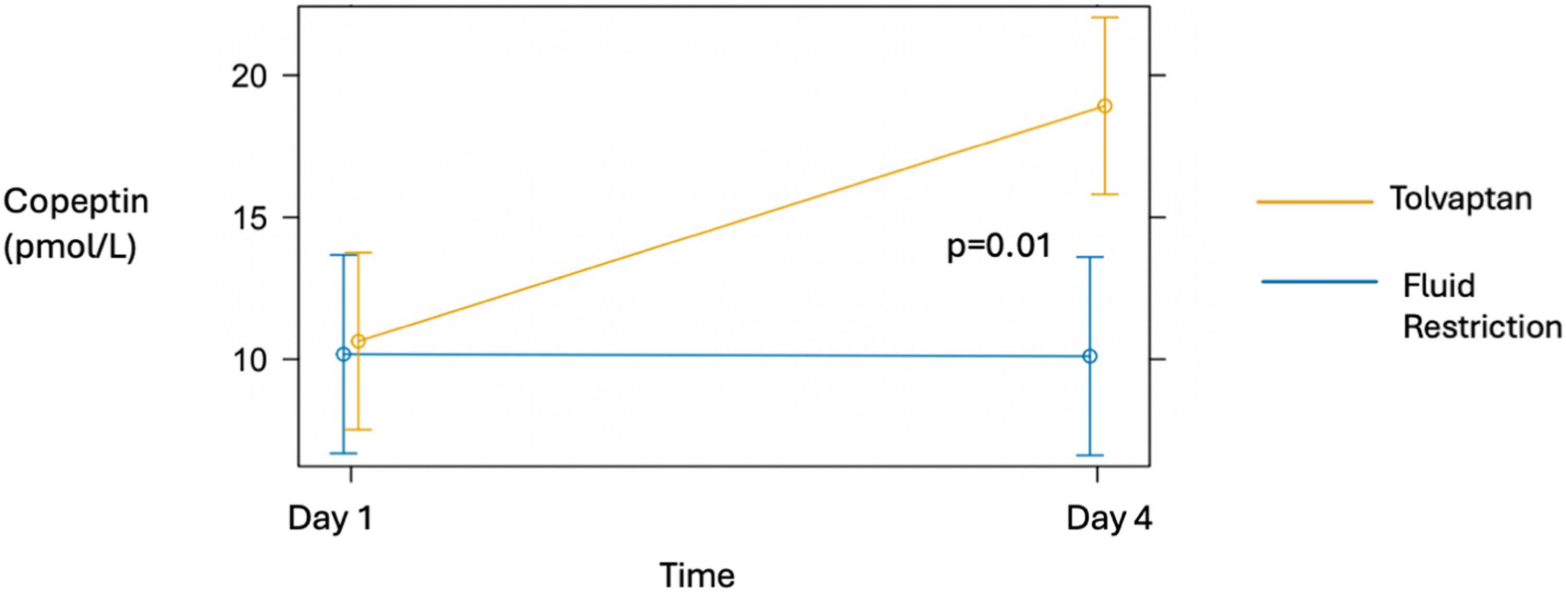
Effect on plasma copeptin of treatment of hyponatraemia with tolvaptan versus fluid restriction over 3 days, with levelled baseline to isolate treatment effect.

Copeptin remained stable with FR over 3 days (−0.07, 95% CI −1.36 to 1.12, *p* = 0.91) but significantly increased in the tolvaptan group over FR at Day 4. The mean adjusted difference in copeptin between groups at Day 4 was 8.4 pmol/L (95% CI 2.1–14.6, *p* = 0.01) (Figure 1). The figure is presented with a levelled baseline to isolate treatment effect, as is customary for an RCT.

Plasma sodium increased over 3 days in both arms, significantly more with tolvaptan than FR (mean adjusted difference 2.5 mmol/L, *p* < 0.001), as previously reported [11]. Serum osmolality also increased to a greater extent in the tolvaptan group (mean adjusted difference 6.9 mOsm/kg, *p* < 0.001)

Rapid sodium rise requiring additional hypotonic fluid to prevent overcorrection occurred in four tolvaptan‐treated patients with copeptin measured (16%). Administration of 5% dextrose according to our protocol ensured no cases of pNa rise above 10 mmol/L/24 h.

There was no association between baseline copeptin concentration (*p* = 0.63) or delta copeptin (*p* = 0.48) and rapid pNa rise following tolvaptan. As previously reported, the only predictor of above‐target rise was lower baseline pNa.

## 4 Discussion

In this pre‐specified secondary analysis of a randomised trial, in 45 hospitalised patients with hyponatraemia predominantly due to SIAD, serum copeptin increased after 3 days of tolvaptan, but remained unchanged with FR, despite rises in plasma sodium and osmolality in both groups (greater with tolvaptan). These findings suggest that AVP‐V2 antagonism may induce compensatory AVP release, reflected by increased copeptin. However, this compensatory response is insufficient to counteract the more effective correction of hyponatraemia seen with tolvaptan compared to FR.

Our mean baseline copeptin concentration was comparable to that previously reported in a larger cohort of 106 patients with SIAD (10.4 vs. 11.9 pmol/L) [18]. Inconsistent with our hypothesis, higher baseline copeptin did not predict rapid pNa rise following tolvaptan. This finding does not support the use of baseline copeptin for risk stratification. To date, lower baseline pNa is the only consistent predictive factor for rapid correction in response to tolvaptan [11, 19, 20].

To our knowledge, this is the first report of increased copeptin following tolvaptan treatment for hyponatraemia. However, copeptin increases have been observed during tolvaptan therapy for other indications, including autosomal dominant polycystic kidney disease [21] and heart failure [22]. This supports the idea that blockade of the AVP‐V2 receptor leads to compensatory increase in AVP and therefore copeptin.

Other SIAD medications have also been associated with rising copeptin. Empagliflozin increased copeptin by approximately 25% (from median 2.6 to 3.8 pmol/L) after 4 weeks in a small outpatient study in SIAD [9]. In contrast, we recorded a 63% rise in copeptin after only 3 days of tolvaptan. This apparent larger effect could be due to tolvaptan's more potent mechanism of direct AVP receptor blockade, though the significant hyponatraemia in our cohort (mean pNa 123 mmol/L) compared to that trial (mean pNa 131 mmol/L) may also be a factor. Data for urea are limited to healthy volunteers with normal sodium, in whom copeptin more than doubled from baseline several hours after administration (4.6–10.1 pmol/L) [10]. Prospective studies directly comparing the different therapies for SIAD would clarify whether copeptin response differs by mechanism of action.

An alternative explanation for rising copeptin with SIAD therapies is that these patients exhibit an exaggerated AVP response to increasing sodium and osmolality (‘reset osmostat’). However, this would not explain why copeptin did not rise following FR in our trial despite increase in sodium and osmolality. A lack of copeptin response to FR aligns with prior data from patients with SIAD in the FR arm of a small 4‐week trial [9].

Furthermore, studies examining copeptin response after hypertonic saline in hyponatraemia have not consistently demonstrated increases in copeptin with sodium correction [1, 23, 24]. In fact, five distinct patterns of copeptin response have been observed, likely reflecting differing underlying etiologies of SIAD [1, 23]. In a trial of acute treatment of patients with severe symptomatic hyponatraemia, mean copeptin *decreased* after 24 h following sodium rise—potentially reflecting falling AVP with improvement in underlying SIAD caused by acute illness and stress [24]. Collectively, the data suggest sodium and osmolality elevation alone do not uniformly increase AVP and copeptin.

Another potential explanation for the rise in copeptin following tolvaptan may be that a more rapid aquaresis from tolvaptan could cause hypotension and therefore volume stimulus to AVP secretion via activation of baroreceptors. It may have been of interest to assess plasma renin activity or blood urea nitrogen to investigate this further, however, these were not measured in our study.

Limitations of our study include modest sample size, particularly with respect to patients with rapid sodium rise. As a secondary (albeit pre‐specified) biomarker analysis, this study was not specifically powered to detect difference in copeptin. The findings should therefore be considered exploratory. Copeptin was measured from frozen serum, which may have impacted assay precision. In addition, we measured copeptin at only two timepoints 3 days apart, so very early or transient changes in copeptin may not be detected. Further limitations include the open‐label design after randomisation, and lack of adjustment for sodium or osmolality.

Strengths include the prospective randomised design in a clinically relevant population of hospitalised patients with moderate‐profound hyponatraemia, predominantly due to SIAD.

To conclude, in the context of an existing trial in patients with hyponatraemia due to SIAD, we found that copeptin rose after treatment with tolvaptan but not FR. Copeptin measurement is not currently routine in hyponatraemia as it does not assist in diagnosis of common causes, or guide treatment. Urine osmolality and urine sodium are measured instead to approximate AVP action.

Further research would be required to determine a clinical utility for measuring copeptin in hyponatraemia before it is adopted into routine practice.

## Ethics Statement

The trial received ethics approval from Austin Health Human Research Ethics Committee (HREC/48055/Austin‐2019).

## Conflicts of Interest

A.M.W., M.G. and N.R. have received a grant‐in‐aid from Otsuka Pharmaceutical Australia for this investigator‐initiated trial. Unrelated to this work, M.G. received research funding from Bayer Pharma, Novartis, Weight Watchers, Lilly and speaker's honoraria from Bayer Pharma and Besins Healthcare.

## Supporting information


Supporting File 1

Supporting File 2

## Data Availability

De‐identified individual‐level data may be shared upon reasonable request, following completion of a signed data agreement.
