# Supplementary material for: Serum Copeptin Rises After Tolvaptan for Hyponatraemia, but Does Not Predict Risk of Rapid Sodium Rise: Pre‐Specified Secondary Analysis of the TVFR Trial
Source: Clin Endocrinol (Oxf). 2026 Aug 3;105(4):514–8. doi: 10.1111/cen.70184 (PMC13532165; doi:10.1111/cen.70184)
Supplement: Supplementary file 1 [file CEN-105-514-s002.docx]

18/06/2026

18/06/2026

18/06/2026

18/06/2026

18/06/2026

Annabelle M Warren

Kay Weng Choy

Rudolf Hoermann

Nicholas Russell

Mathis Grossmann

Serum copeptin rises after tolvaptan for hyponatremia, but does not predict risk of rapid sodium rise: Pre-specified secondary analysis of the TVFR trial.
