# Supplementary material for: Serum Copeptin Rises After Tolvaptan for Hyponatraemia, but Does Not Predict Risk of Rapid Sodium Rise: Pre‐Specified Secondary Analysis of the TVFR Trial
Source: Clin Endocrinol (Oxf). 2026 Aug 3;105(4):514–8. doi: 10.1111/cen.70184 (PMC13532165; doi:10.1111/cen.70184)
Supplement: Supplementary file 2 [file CEN-105-514-s001.doc]

**CONFLICT OF INTEREST**

**Paper Number: ______** **2037855_________**

**Name: _______Dr Annabelle M Warren________________**

As corresponding author of the above manuscript which has been accepted for publication in *Clinical Endocrinology,* please list below all co-authors stating whether or not they have any possible conflicts of interest. Only conflicts of interest specific to this manuscript need be declared. Please return the completed form to the Editorial Office (cenedoffice@wiley.com).

The following are examples of possible conflicts of interest:

1. Source of funding
2. Paid consult to sponsor
3. Study investigator funded by sponsor
4. Employee of sponsor
5. Board membership with sponsor
6. Stock holder for mentioned product
7. Patent inventor for mentioned product
8. Any financial relationship to competitors of mentioned product

This information will be kept confidential. The Editor will discuss the method of disclosure of any potential conflict of interest with corresponding authors on an individual basis.

*Author	No Conflict	Conflict (Please specify)*

Annabelle M Warren	Received a grant-in-aid from Otsuka Pharmaceutical Australia for this investigator-initiated trial

Kay Weng Choy	X

Rudolf Hoermann	X

Nicholas Russell	Received a grant-in-aid from Otsuka	Pharmaceutical Australia for this

investigator-initiated trial

Mathis Grossmann	Received a grant-in-aid from Otsuka	Pharmaceutical Australia for this

investigator-initiated trial

Unrelated to this work, MG received research funding from Bayer Pharma, Novartis, Weight Watchers, Lilly and speaker’s honoraria from Bayer Pharma and Besins Healthcare.
